# Host-driven evolution of PCV2: insights into genetic diversity and adaptation

**DOI:** 10.3389/fimmu.2025.1577436

**Published:** 2025-05-26

**Authors:** Giovanni Franzo, Matteo Legnardi, Francesca Poletto, Riccardo Baston, Mattia Cecchinato, Michele Drigo, Claudia Maria Tucciarone

**Affiliations:** Department of Animal Medicine, Production and Health, University of Padova, Padua, Italy

**Keywords:** porcine circovirus 2 (PCV2), evolution, phylodynamic, selection, capsid, attachment, amino acids

## Abstract

**Introductions:**

Porcine circovirus type 2 (PCV2) is a significant pathogen in swine, exhibiting notable genetic and phenotypic diversity. This study explores the evolutionary mechanisms influencing PCV2 variability, emphasizing the role of viral features and host environment.

**Methods:**

An extensive collection of globally available ORF2 sequences of the main PCV2 genotypes (i.e., PCV2a, PCV2b, and PCV2d) sampled from wild boars and domestic pigs was analyzed, using a combination of phylodynamic approaches and biostatistical methods to infer the presence and patterns of selective pressures in different virus population subsets.

**Results:**

Significant differences were observed between strains collected from domestic and wild populations, with the former appearing to be under stronger selective pressures at specific capsid positions. These pressures are likely driven by immune-mediated selection acting on critical residues for immune system recognition and evasion. A comprehensive evaluation of substitution patterns also revealed a trend toward maintaining or enhancing amino acid polarity, with neutral residues often replaced by polar or charged ones. This shift may influence the virus interaction with host proteins, particularly glycosaminoglycans such as heparan sulfate-like molecules. The observed variability among hosts and genotypes highlights both the importance of host environment as a key driver of viral evolution and the plasticity of PCV2 adaptability, with multiple alternative evolutionary pathways seemingly being selected.

**Discussion:**

The findings underscore the complex evolutionary trajectories followed by PCV2 on a global scale and suggest that the intensification of pig farming and associated management practices may have significantly shaped PCV2 evolution, contributing to the current epidemiological landscape.

## 1 Introduction

Porcine circovirus 2 (PCV2) was initially identified in the 1990s and quickly emerged as one of the most significant pathogens affecting intensive pig farming (1). The causative agent, classified in the species *Circovirus porcine 2*, genus *Circovirus*, is a non-enveloped virus characterized by a single-stranded circular genome of approximately 1.7 kb (2). Different open reading frames (ORFs) have been predicted, and the expression of at least six proteins has been proven and investigated. Among these, two are pivotal for viral replication: ORF1, encoding the proteins Rep and Rep’ through alternative splicing, which are involved in rolling-circle replication, and ORF2, coding for the protein Cap, the sole constituent of the viral capsid (3). ORF2 and its corresponding protein are by far the most studied due to their biological and epidemiological relevance. Besides mediating viral attachment to the cell and participating in other important steps of the viral cycle, Cap is the main target of the host immune response. For this reason, it is the most variable region and is commonly used for molecular epidemiological studies and classification purposes (4). Other ORFs (ORF3–6) are transcribed and translated, playing essential roles in apoptosis regulation and interference with host signaling pathways at various levels (5–8). PCV2 exhibits a remarkable evolutionary rate (about 10^3^³–10^34^ substitutions per site per year), which is at the upper limits for single-stranded DNA viruses (9, 10). Additionally, a high recombination rate has been reported, with the most likely breakpoints occurring in the intergenic regions (11, 12). This has led to the emergence of several variants over time, characterized by significant genetic variability, and classified into genotypes.

Currently, eight genotypes have been formally defined (PCV2a–h) (13) based on ORF2 phylogenetic and genetic distance analyses, and another genotype has been proposed (14). Of these, three—PCV2a, PCV2b, and PCV2d—are considered major genotypes due to their persistent worldwide distribution. PCV2a was the first identified and remained the most prevalent genotype until around 2003, when it was replaced by PCV2b. Around 2010, PCV2b was supplanted by PCV2d, which is currently the dominant genotype (10). However, recent evidence has shown that PCV2a and PCV2b have experienced several waves of resurgence and decline, continuing to circulate and evolve over time, even as PCV2d remains predominant (15).

In intensively raised animals, PCV2 infection has been associated with postweaning multisystemic wasting syndrome (PMWS, now known as PCV2 systemic disease or PCV2-SD), PCV2 reproductive disease (PCV2-RD), and porcine dermatitis and nephropathy syndrome (PDNS). These three clinical entities are collectively referred to as porcine circovirus diseases (PCVDs) (16). The pathogenesis of PCVDs is multifactorial and influenced by factors typical of intensive pig farming, such as increased infective pressure, reduced animal responsiveness, and enhanced within-host viral replication, leading to overt clinical signs (17). Although most infections remain subclinical (PCV2 subclinical infection, PCV2-SI), they still cause major economic losses due to reduced productive performance (17). The advent of vaccines revolutionized the scenario, providing remarkable protection from clinical syndromes and improving performance even in subclinically affected animals (18). Despite these benefits, some evidence suggests variable cross-protection among genotypes, which may have conferred a competitive advantage to certain genotypes, particularly PCV2d (4). Moreover, the selective pressures imposed by vaccine-induced immunity appear to have played a role in viral evolution (19, 20).

Beyond intensively raised pigs, PCV2 has been reported in several other host species, demonstrating a broader-than-expected tropism (21). While the epidemiological relevance in most of these species is likely negligible, this is not the case for wild boar populations. Various studies have reported a significant PCV2 prevalence in wild boars and their ability to sustain the virus through extensive transmission chains (22–24). Contact with intensively raised pigs has also been demonstrated, potentially mediated by rural and backyard pig production systems (24). Interestingly, wild boars are typically not affected by PCVDs, which might reflect differences in host features or adaptations, or more likely, the absence of co-factors characteristic of intensive pig farming. Additionally, wild boar populations are not vaccinated, providing a valuable opportunity to investigate the potential effects of vaccine-induced pressures on PCV2 evolution by comparing the selective pressures acting on domestic and wild populations.

To investigate how viral circulation among domestic and wild populations influences viral evolution, we analyzed a globally available collection of PCV2 sequences. We used several approaches to assess the roles of these hosts in maintaining the viral population, to evaluate the exchange between them, and to identify differential selective pressures and evolutionary patterns in various host environments.

## 2 Materials and methods

### 2.1 Sequence selection

Complete PCV2 ORF2 sequences were downloaded from GenBank (accessed on 17/09/2024), when information on collection host, country, and year was available. To identify sequences collected from wild boar, the string “wild” was iteratively searched in the features field of each GenBank record, and the relevant sequences were extracted. Instances where classification was uncertain were manually verified by reviewing the associated article when available; otherwise, they were excluded from the analysis. Selection was primarily based on wild boar sequences, and thereafter domestic pig sequences were matched aiming to achieve a 1:5 ratio using the annotated sequences provided in the Franzo et al., 2024 dataset (15). This ratio was based on a preliminary structured coalescent analysis (see Section 2.3) performed to estimate the viral population size circulating in wild boar and domestic pig populations, using an initial dataset that included the respective sequences in a 1:1 ratio. However, matching was also performed to achieve a comparable temporal and spatial distribution (at least at the continent level). When not enough sequences were available in the considered year, all the present ones were included. Moreover, pairwise genetic distances among strains were calculated and compared between the two populations to ensure a comparable distribution. This was done to verify that the phenotypic variability under investigation, i.e. variation in the amino acid profile, was not merely a reflection of genetic variability.

### 2.2 Strain classification and dataset generation

Selected ORF2 sequences, along with the references provided by Franzo et al., 2018 (13) were aligned at the amino acid level and then back-translated to the nucleotide level using the MAFFT (25) algorithm implemented in TranslatorX (26). Sequences showing premature stop codons, frameshift mutations, or poor alignment were excluded from further analysis. Recombination occurrence was assessed using RDP5 (27). Sequences identified as recombinant by at least three methods with a p-value < 10^-5^ after Bonferroni correction were removed. Specifically, RDP, GENECONV, Chimaera, and 3Seq were used for the primary scan, while the full set of available methods was employed for analysis refinement. The RDP5 settings for each method were adjusted to account for the dataset characteristics, following the RDP manual recommendations. For genotyping, a phylogenetic tree was reconstructed using IQ-TREE (28), selecting the substitution model with the lowest Bayesian Information Criterion (BIC). The reliability of the inferred clades was assessed by performing 1,000 bootstrap replicates. Besides the overall dataset, including strains belonging to genotype PCV2a, b and d (PCV2abd), genotype specific (PCV2a, PCV2b and PCV2d) dataset were generated. These datasets were also split into wild boar and domestic pig specific dataset.

### 2.3 Structured coalescent

The viral effective population size (Ne) and migration rates among host categories (Domestic pig and Wild boars) were estimated simultaneously using Bayesian structured coalescent analysis, implemented in the Mascot package (29) of BEAST 2.7 (30). A Markov chain Monte Carlo (MCMC) run of 100 million generations was performed on dataset PCV2abd, PCV2a, PCV2b and PCV2d, with model parameters and trees sampled every 10,000 generations. The nucleotide substitution model was selected based on the BIC, and a relaxed lognormal molecular clock was applied. Results were analyzed with Tracer 1.7 and were considered reliable if the estimated sample size (ESS) exceeded 200 and convergence and mixing were satisfactory after discarding 20% of the samples as burn-in. Parameter estimates were summarized as means and 95% highest posterior density (HPD) intervals. Maximum clade credibility (MCC) trees were constructed and annotated using TreeAnnotator from the BEAST package.

### 2.4 Selective pressure analysis

Different methods based on the estimation of the non-synonymous to synonymous substitution rate were evaluated to investigate potential differences in the selective pressures acting on viruses sampled from different host populations. Briefly, the ratio (or equivalently the difference) of non-synonymous (β) to synonymous (α) substitution rates (ω) can be used to infer evolutionary pressures, with ω < 1 indicating purifying (negative) selection where most non-synonymous changes are deleterious and removed by natural selection, ω = 1 suggesting neutral evolution where non-synonymous and synonymous substitutions occur at similar rates with no selection, and ω > 1 reflecting diversifying (positive) selection where non-synonymous changes are favored due to their selective advantage. The occurrence of episodic diversifying selection was estimated using MEME (Mixed Effects Model of Evolution) method (31) on host-specific dataset. Analyses were performed on all available sequences and on genotype specific dataset. The occurrence of different selective regimes acting on the sets of branches the phylogenetic trees corresponding wild and domestic pigs was tested using Contrast-FEL (32). Contrast-FEL allows to examine selective pressures (measured as β/α) individually at each site in the gene, and test whether or not they are different between environments. It must be stressed that a significant result at a given site indicates that the β/α ratio differs between the two sets of branches, reflecting either an increase or decrease in the test branches compared to the reference branches. However, a significant result does not imply whether the selection is positive (β/α > 1) or negative (β/α < 1); it indicates that the β/α ratio varies between the branch sets, without necessarily altering the mode of selection.

Similarly, the presence of directional selective pressures (i.e. the tendency to mutate toward specific amino acids) was tested using FADE (FUBAR Approach to Directional Evolution) (33). Specifically, FADE systematically test, for each site in the alignment, whether a specified set of foreground branches shows a substitution bias towards a particular amino acid, compared to background branches. In both instances, genotype specific phylogenetic trees were reconstructed using IQ-Tree and the branches were labelled using phylotree (https://phylotree.hyphy.org/). Terminal branches corresponding to domestic pigs were labelled as foreground, while for internal one labelling, a maximum parsimony approach was applied. Wild boar branches were considered as background.

PRoperty Informed Models of Evolution Protein (PRIME) method was used to assess the role of chemical-physical properties in influencing the amino acid substitutions. PRIME allows the non-synonymous substitution rate β depending not only on the site in question, but also on which residues are being exchanged. In particular, the amino acid properties were coded using the Atchley et al. (34) scale, as implemented in Hyphy (35). Briefly, the approach attempts to identify codon positions where substitutions leading to amino acid exchanges that result in a radical change in the property of interest (e.g., a significant variation in isoelectric point) occur at an accelerated rate compared to substitutions causing conservative changes (e.g., amino acids with a similar isoelectric point). The significance level was set at p < 0.05.

## 3 Results

### 3.1 Dataset

A total of 2615 sequences were included in the final dataset, collected from 50 countries and spanning a time interval from 2002 to 2022. Of these, 469 were collected from wild boars and 2146 from domestic pigs. Two hundred and eighty-five were classified as PCV2a, 802 as PCV2b, and 1528 as PCV2d (**Supplementary Table 1**).

### 3.2 Structure coalescent analysis

**Figure 1:**
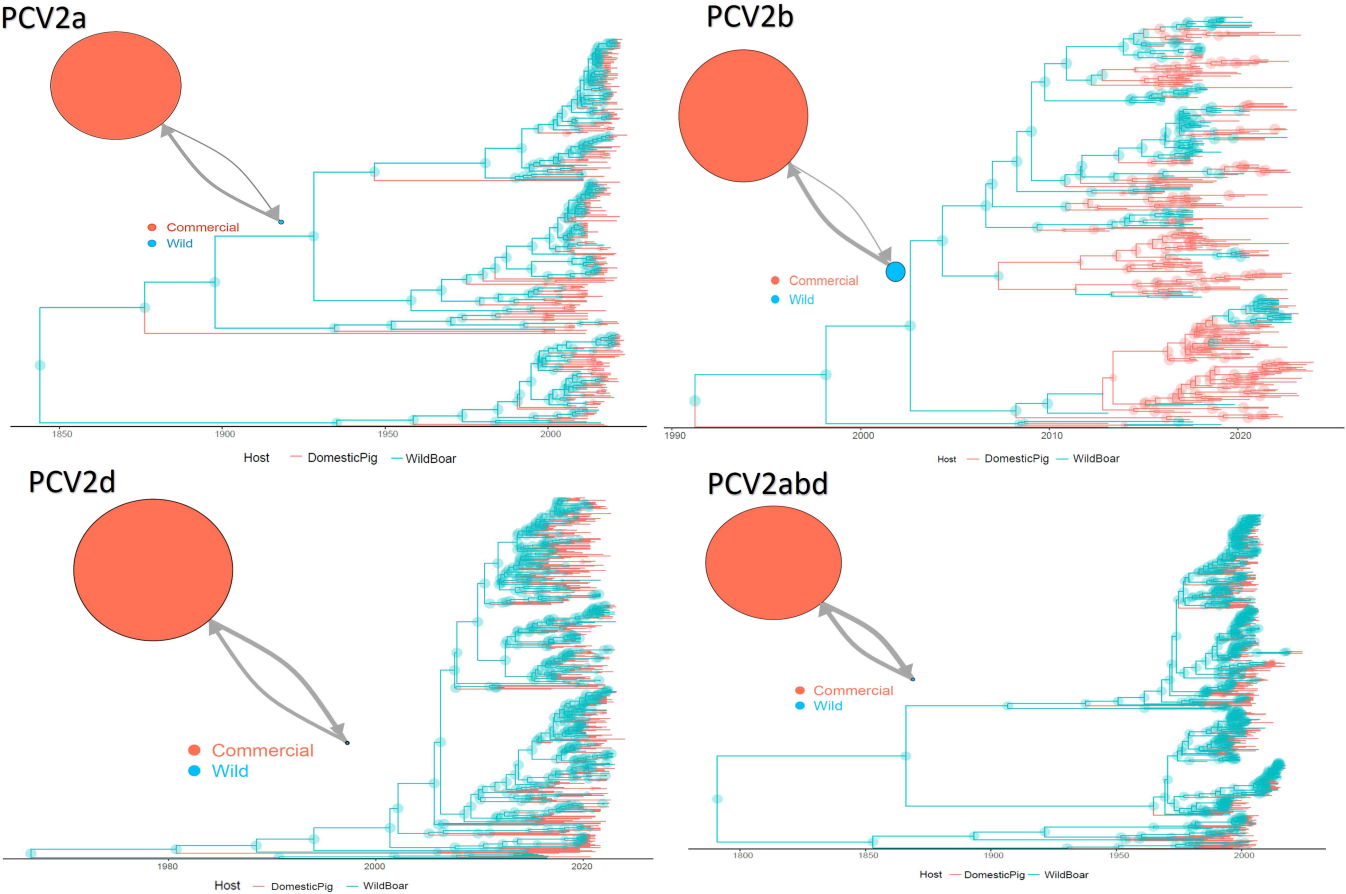
Structured coalescent-based phylogenetic tree of PCV-2a, b, d and abd dataset. Branch colors, as from the legend, mark the inferred animal category where the ancestral strain was circulating, while node size represents the posterior confidence of the inference. The network on the upper left of each panel depicts the migration rate between animal categories. Arrows and circle sizes are proportional to the inferred migration rate and population size, respectively.

The tMRCA of PCV2a was estimated in 1843.42 [95HPD:1755.21-1917.42] with an estimated evolutionary rate of 4.48·10–^4^ [95HPD:3.11·10^-4^ -5.85·10^-4^]. The population size of domestic pigs was inferred about 25 time bigger than the wild one, while the migration rate from wild to domestic was approximately twice the opposite one (**Figure 1**). The tMRCA of PCV2b was estimated in 1957.77 [95HPD:1937.55-1992.08] with an estimated evolutionary rate of 7.34·10–^4^ [95HPD:5.31·10^-4^ -9.55·10^-4^]. The population size of domestic pigs was inferred about 7 times bigger than the wild one, while the migration rate from wild to domestic was approximately three-times the opposite one (**Figure 1**). The tMRCA of PCV2d was estimated in 1964.86 [95HPD:1935.02-1989.83] with an estimated evolutionary rate of 5.35·10–^4^ [95HPD:3.64·10^-4^ -7.14·10^-4^]. The population size of domestic pigs was inferred about 70 times bigger than the wild one, while the migration rate from wild to domestic was approximately three-times the opposite one (**Figure 1**). Finally, the tMRCA of PCV2abd was estimated in 1796.43 [95HPD:1678.85-1883.75] with an estimated evolutionary rate of 5.35·10–^4^ [95HPD:3.64·10^-4^ -7.14·10^-4^]. The population size of domestic pigs was inferred about 40 times bigger than the wild one, while the migration rate from wild to domestic was approximately two and a half times the opposite one (**Figure 1**). In all datasets, a wild boar origin was estimated.

### 3.3 Selective pressure analysis

**Figure 2:**
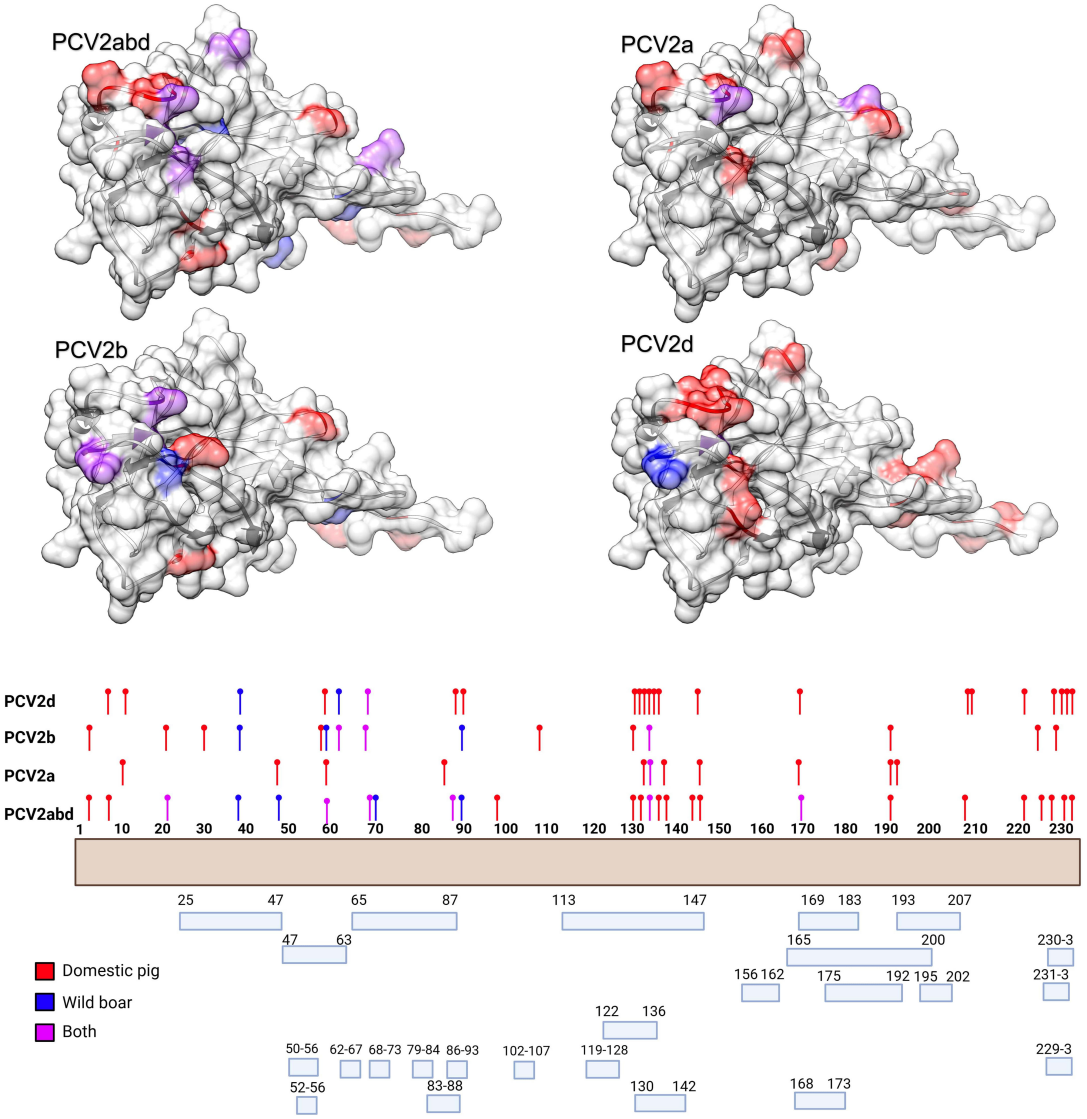
In the upper panel, the amino acid positions under statistically significant diversifying selection, as identified by MEME, are mapped onto the tertiary structure of PCV2 capsid protein. Different figures are used to represent the various datasets analyzed. Host species (or combinations of hosts) are color-coded. In the lower panel, a summary of the distribution of the same amino acid positions along the Cap sequences is presented, alongside the epitopic regions identified in previous experimental studies (36–41).

**Figure 3:**
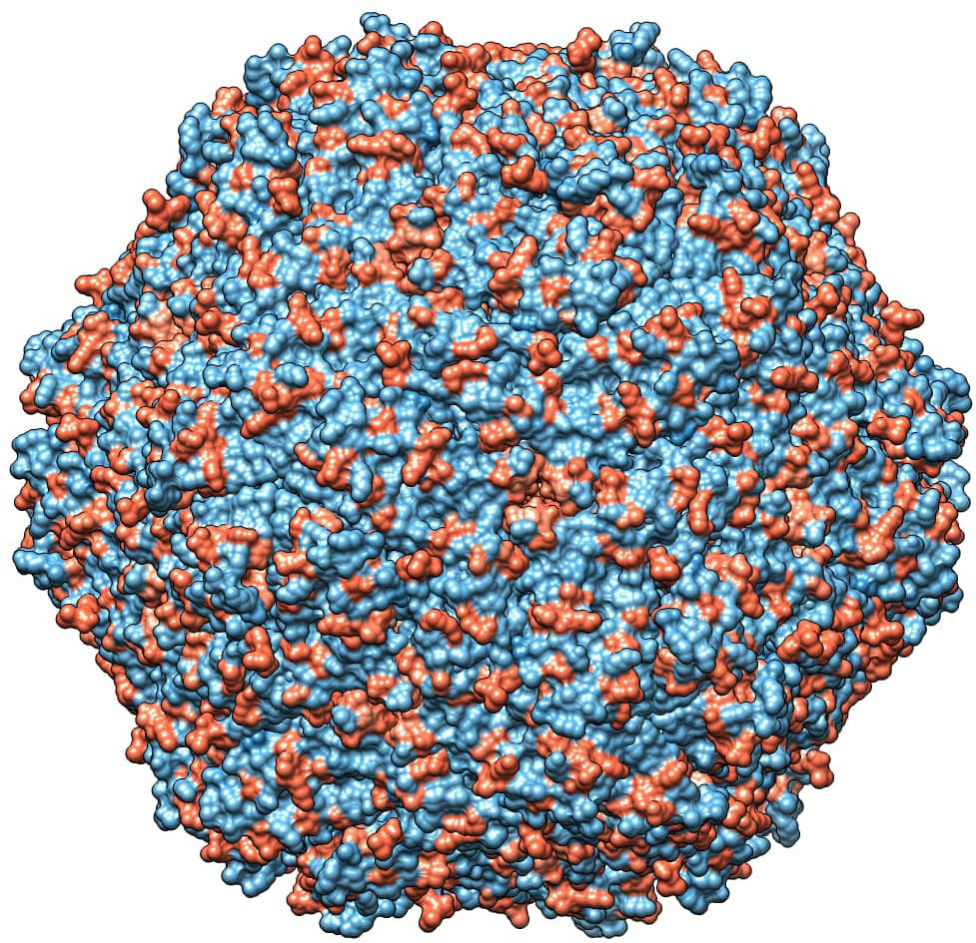
Depiction of the contrast-FEL results. Sites with a higher non-synonymous substitution rate (β) in wild boars and domestic pigs are depicted in blue and red, respectively. Both statistically significant and non-significant sites are reported.

**Figure 4:**
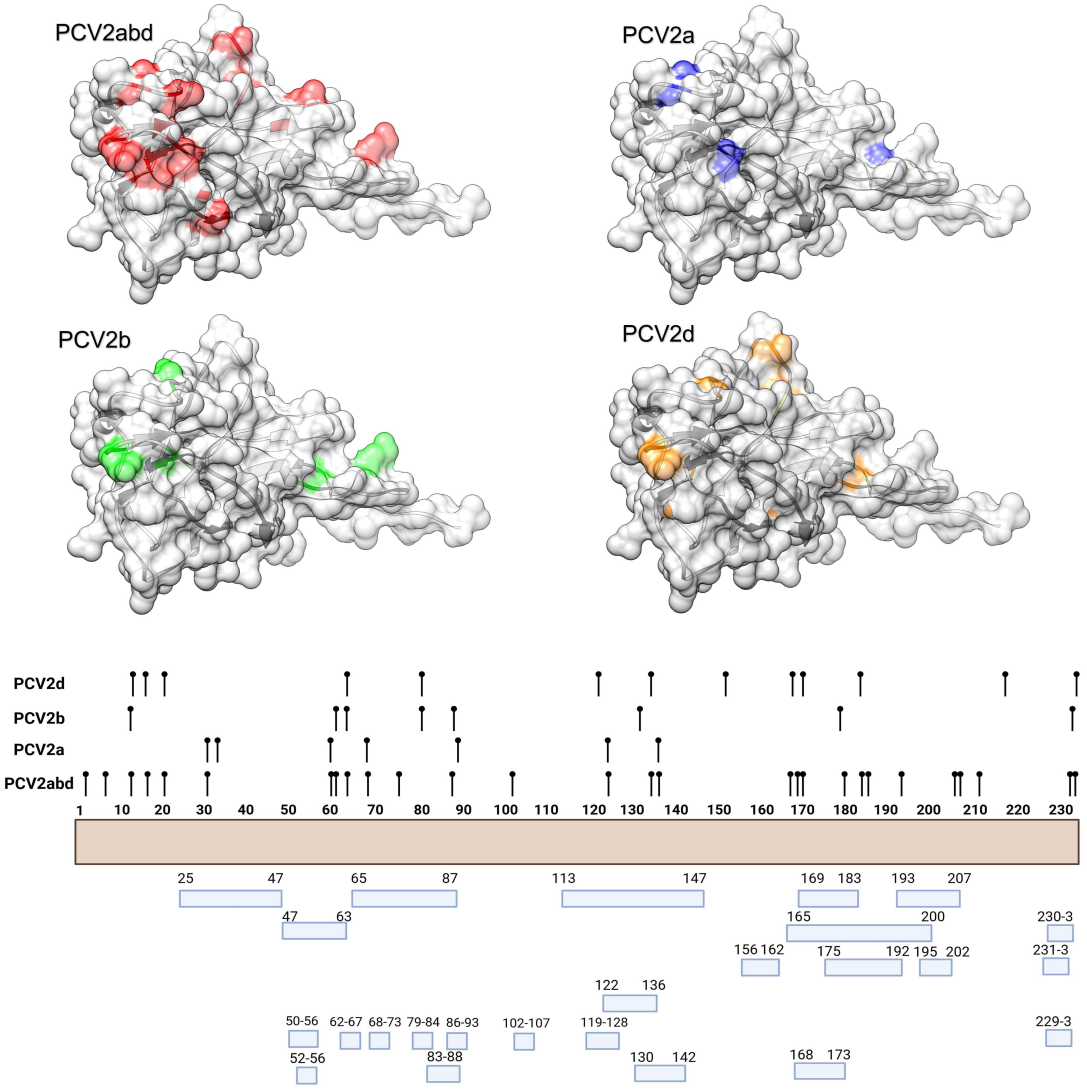
In the upper panel, the amino acid positions under statistically significant directional selection, as identified by FADE, are mapped onto the tertiary structure of PCV2 capsid protein. Different figures are used to represent the various datasets analyzed. In the lower panel, a summary of the distribution of the same amino acid positions along the Cap sequences is presented, alongside the epitopic regions identified in previous experimental studies (36–41).

The MEME analysis identified 26 amino acid positions under episodic diversifying selection in the PCV2abd dataset: of those, 16 were found in domestic pigs, 4 in wild boars, and 6 in both datasets. In the PCV2a dataset, 9 positions were statistically significant in the domestic pig dataset and 2 in both datasets. Fifteen positively selected positions were also identified in the PCV2b dataset: 9 in domestic pigs, 3 in wild boars, and 3 in both sequence sets. Finally, 23 positions were identified for PCV2d, with 20 of those in domestic pigs, 2 in wild boars, and 1 in both datasets. A summary of the genotype- and host-specific positions is provided in **Table 1** and **Figure 2**. Regardless of the host species, most of the detected amino acid positions were exposed on the capsid surface, in areas previously experimentally proven to be epitopic regions (**Figure 2**). Among the sites identified by MEME, positions 8, 59, 90, 133, 134, and 234 were found to be under differential selective pressures among hosts using Contrast-FEL. Overall, a higher β (non-synonymous substitution rate) was inferred for wild boar strains, both at statistically significant and non-significant sites. However, in specific residues, particularly those exposed to the external environment, the opposite trend was observed (**Figure 3**). The FADE analysis, targeting residues experiencing directional selective pressures following the introduction from wild to domestic populations, identified 27 sites in the complete PCV2abd dataset, 7 in PCV2a, 8 in PCV2b, and 13 in PCV2d (**Table 2**, **Figure 4**).

**Table 1:** Summary of the codon position where statistically significant evidence of episodic diversifying selection was identified.

*Codon*	*PCV2a*	*PCV2b*	*PCV2d*	*PCV2abd*
*3*	–	Domestic	–	Domestic
*8*	–	–	Domestic	Domestic
*12*	–	–	Domestic	–
*13*	Domestic	–	–	–
*21*	–	Domestic	–	Both
*30*	–	Domestic	–	–
*39*	–	Wild	Wild	Wild
*47*	Domestic	–	–	Wild
*58*	–	Domestic	–	–
*59*	Domestic	Wild	Domestic	Both
*63*	–	Both	Wild	–
*68*	–	Both	Both	Both
*70*	–	–	–	Wild
*86*	Domestic	–	–	–
*88*	–	–	Domestic	Both
*89*	–	–	Domestic	–
*90*	–	Wild	Domestic	Wild
*108*	–	Domestic	–	Domestic
*130*	–	Domestic	–	Domestic
*131*	–	–	Domestic	–
*133*	Domestic	–	Domestic	Domestic
*134*	Both	Both	Domestic	Both
*135*	–	–	Domestic	Domestic
*136*	–	–	Domestic	–
*137*	Domestic	–	–	Domestic
*144*	–	–	Domestic	Domestic
*146*	Domestic	–	–	Domestic
*169*	Domestic	–	Domestic	Both
*190*	Domestic	Domestic	–	Domestic
*191*	Both	–	–	–
*208*	–	–	Domestic	–
*209*	–	–	Domestic	Domestic
*222*	–	–	Domestic	Domestic
*224*	–	Domestic	–	Domestic
*228*	–	Domestic	Domestic	Domestic
*230*	–	–	Domestic	–
*233*	–	–	Domestic	Domestic
*234*	–	–	Domestic	Domestic

The considered genotype datasets are reported in different columns while the involved host species are reported in the cells.

**Table 2:** Summary of amino acid positions detected under episodic directional selection by FADE, applied to different datasets with wild boars as the background and domestic pigs as the foreground branches.

*Codon*	*PCV2a*	*PCV2b*	*PCV2d*	*PCV2abd*
*2*	–	–	–	*T*->M (3)**R** (1)
*8*	–	–	–	F->I (1)L(3)*T*(1)*Y*(15), *Y*->F(7)
*11*	–	**R**->G(1)**K**(5)	–	–
*12*	–	–	**R**->I(6)**K**(2)*T*(1)	**R**->I(8)**K**(2)*S*(1)*T*(1)
*16*	–	–	R->C(3)H(1)P(1)	**R**->C(3)G(1)**H**(2)P(1)
*20*	–	–	G->A(1)*S*(3)V(1)	G->A(1)*S*(5)V(1)
*30*	V->I(2)L(8)	–	–	V->A(1)I(5)L(19)
*32*	P->L(2)	–	–	–
*59*	A->**R**(1)*S*(1), **R**->A(2)G(1)	–	–	A->G(1)**K**(2)**R**(5)S(1), G->R(1), **K**->**R**(6), **R**->**K**(1)
*60*	–	*T*->*S*(6)	–	*T*->A(1)S(9)
*63*	–	**K**->**R**(4), **R**->**K**(8)*S*(1), S->**K**(1)	**R**->**K**(5)*S*(1)*T*(1), S->**K**(1)**R**(1)	**K**->**R**(4), **R**->G(1)**K**(8)*S*(2)*T*(2), *S*->G(1)**R**(2), *T*->I(1)**R**(6)*S*(2)
*68*	–	–	–	A->*N*(2)*S*(5)*T*(4), *N*->A(2)**K**(3)*S*(1)
*76*	I->L(5)	–	–	I->L(8)*N*(1)
*80*	–	L->I(6)P(2), V->L(1)	V->L(1)	–
*88*	–	**K**->P(1)	–	**K**->E(2)P(1), P->F(1)
*89*	**R**->I(1)	–	–	–
*101*	–	–	–	V->A(1)I(12)L(1)
*121*	–	–	*S*->*T*(1), *T*->*N*(1)*S*(6)	–
*123*	V->I(8)	–	–	V->A(1)I(11)L(1)
*131*	–	M->*T*(1), *T*->P(9)	–	–
*133*	–	–	A->G(1)H(3)**R**(1)*S*(2)T(3)V(1), **H**->D(1)P(1)*Y*(2), V->A(1), *Y*->F(1)*S*(1)	–
*134*	–	–	–	A->P(1), D->A(1)*N*(1), N->D(11)**K**(4)P(1)*Q*(1)*S*(1)*T*(7), P->*Q*(1), *T*->A(2)I(1)*N*(7)P(10)
*136*	L->Q(3)	–	–	L->F(2)I(1)M(1)*Q*(4)R(2)V(1), M->L(1)
*151*	–	–	P->T(4), *T*->P(2)*S*(1)	–
*166*	–	–	V->I(1)L(5)	V->I(2)L(7)
*169*	–	–	G->A(1)C(1)**R**(16)*S*(2), **R**->G(41)*N*(1)*S*(13)*T*(2), *S*->*N*(1)**R**(2), *T*->**R**(1)	G->A(1)C(1)**R**(8)*S*(1), **R**->G(31)*S*(11)*T*(3), *S*->C(1)*N*(2)P(1)**R**(1), *T*->*N*(1)*S*(1)
*178*	–	N->*S*(6)	–	*N*->*S*(6)*T*(3)
*183*	–	–	L->I(4)	L->I(5)
*185*	–	–	–	L->P(1), M->I(4)L(6)
*191*	–	–	–	A->G(1), G->A(3)E(2)**K**(1)**R**(2)V(1), **R**->**K**(3)
*205*	–	–	–	*S*->G(1)**R**(5)
*206*	–	–	–	I->**K**(4), **K**->I(9)*T*(1)
*210*	–	–	–	D->E(11)G(2)*T*(1), E->D(11)G(1)
*217*	–	–	M->L(12)**R**(1)V(3)	–
*232*	–	**K**->N(2), *N*->I(1)**K**(13)*T*(3)	–	**K**->N(12), *N*->**H**(1)I(1)**K**(37)*T*(6)
*234*	–	–	**K**->F(1)L(1)M(2)N(3)*Q*(5)S(2), L->P(1)	**K**->F(1)L(1)M(3)N(4)P(1)*Q*(6)S(2), M->L(1)

Arrows indicate the directionality of the mutation, and the number of inferred mutations along the phylogenetic tree branches (i.e., viral history) is reported in brackets. Positively charged amino acids—Arginine (R), Lysine (K), and Histidine (H)—are highlighted in bold while polar, non-charged ones are reported in italics.

Directional selection, tested by comparing different host populations, primarily occurred at positions exposed on the surface of the capsid and in regions previously reported as epitopic or involved in viral attachment. Notably, when positively charged amino acids were considered, the directionality was most often toward the acquisition of positive amino acids or their replacement with other positively charged residues. Exceptions to this trend were limited, at least when considering the overall PCV2abd dataset (**Table 2**).

**Figure 5:**
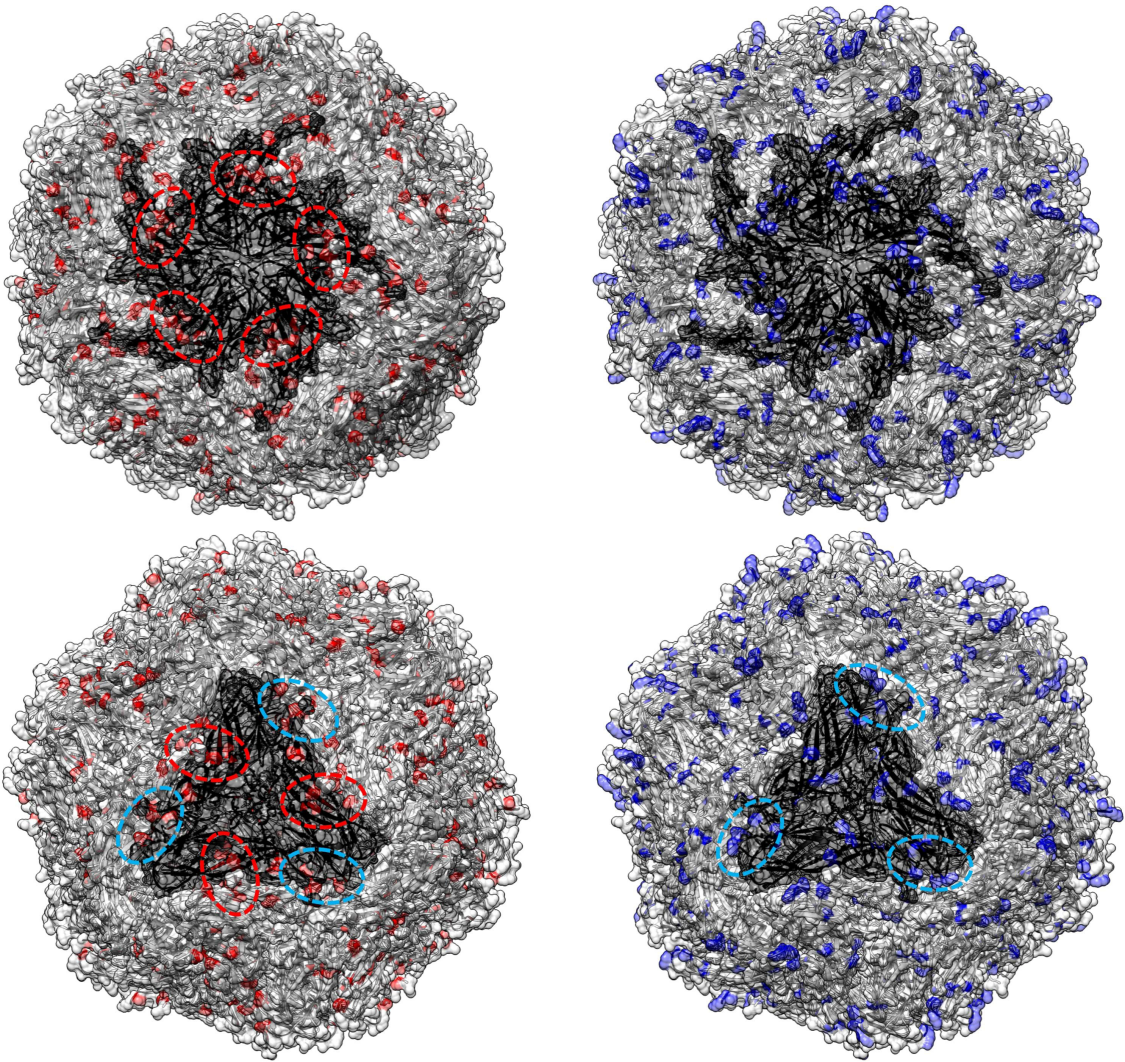
In the left panel, sites with a tendency toward radical changes in amino acid charge, as detected using PRIME in the PCV2abd dataset, are depicted in red. The pentameric and trimeric organization of the PCV2 capsid structure are highlighted in darker gray. In the right panel, positions reported by Dhindwal et al., 2019 (42) as involved in heparan sulfate binding are colored in blue. Dotted red circles emphasize regions identified by Ouyang et al. (2023) (43) as affected by charge modifications, while blue circles highlight additional areas where a comparable distribution of heparan sulfate binding sites and sites under selective pressure were observed in the present study.

**Figure 6:**
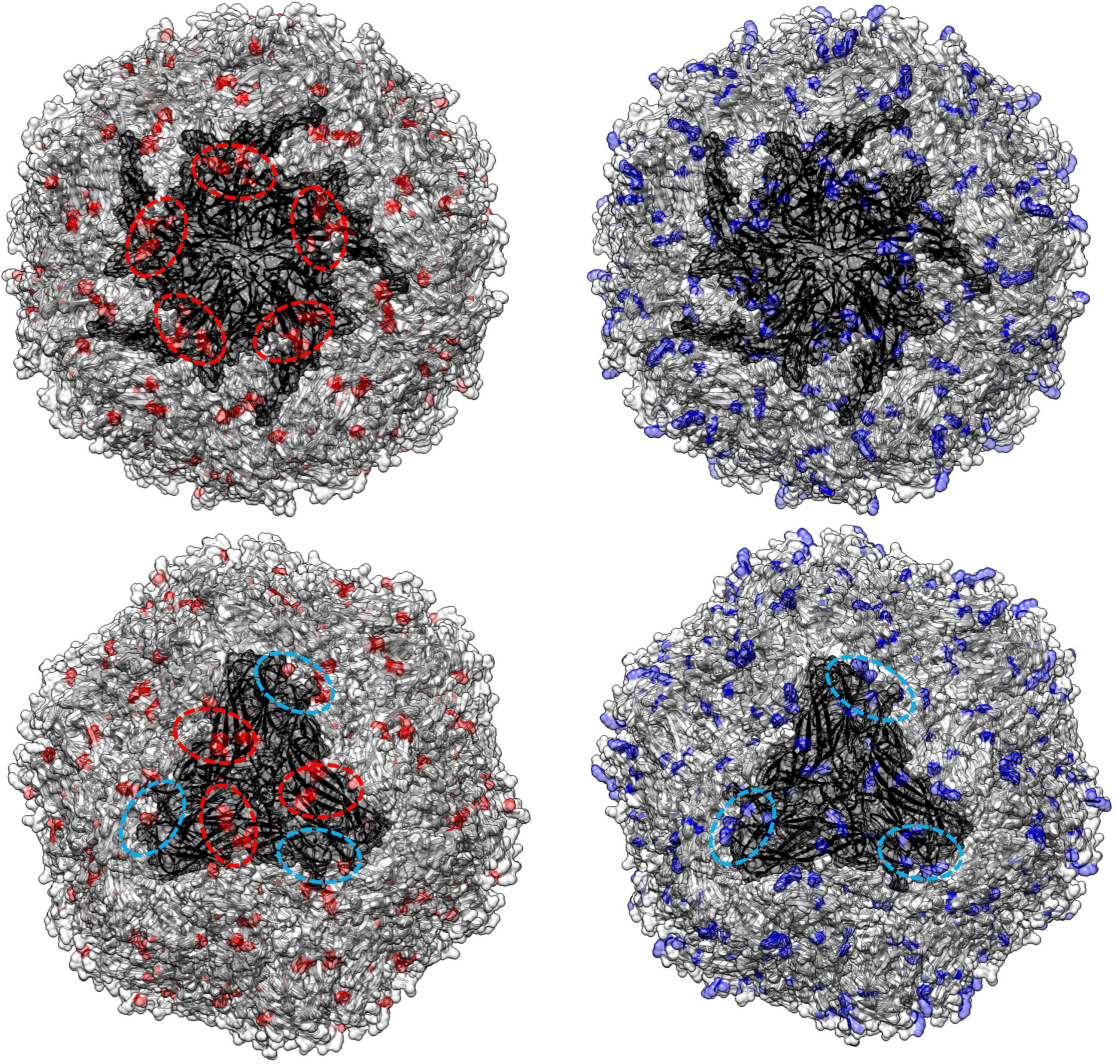
In the left panel, sites with a tendency toward radical changes in amino acid charge, as detected using PRIME in the PCV2d dataset, are depicted in red. The pentameric and trimeric organization of the PCV2 capsid structure are highlighted in darker gray. In the right panel, positions reported by Dhindwal et al., 2019 (42) as involved in heparan sulfate binding are colored in blue. Dotted red circles emphasize regions identified by Ouyang et al. (2023) (43) as affected by charge modifications, while blue circles highlight additional areas where a comparable distribution of heparan sulfate binding sites and sites under selective pressure were observed in the present study.

The role of chemical-physical properties of amino acids, with particular reference to charge, in affecting the tendency to non-synonymous mutations was inferred with PRIME, highlighting a trend toward a radical change in amino acid charge features in 16 positions (15 in domestic and 1 in wild boars) in the PCV2abd dataset, 1 in wild boards in PCV2a, 2 in domestic pigs in PCV2b and 16 in PCV2d (15 in domestic and 1 in both datasets) (**Table 3**). These sites were predominantly exposed on the viral surface and showed a distribution corresponding to amino acid positions reported to be involved in capsid-cell binding mediated by heparan sulfate (HS) and other glycosaminoglycan (GAG) molecules. A focus on PCV2abd and PCV2d is provided due to the higher number of detected sites (**Figures 5**, **6**).

**Table 3:** Summary of the codon position having statistically significant evidence of accelerated substitutions toward radical changes in amino acid charge.

*Codon*	*PCV2a*	*PCV2b*	*PCV2d*	*PCV2abd*
*17*	–	–	Domestic	–
*59*	–	–	Domestic	Domestic
*68*	–	–	Both	Wild
*72*	–	–	Domestic	–
*73*	–	–	Domestic	Domestic
*83*	–	–	–	Domestic
*88*	–	–	–	Domestic
*90*	–	–	Domestic	–
*108*	–	Domestic	–	–
*131*	–	–	–	–
*133*	–	–	–	Domestic
*134*	Wild	Domestic	–	–
*136*	–	–	Domestic	–
*209*	–	–	Domestic	Domestic
*210*	–	–	–	Domestic
*225*	–	–	–	Domestic

The considered genotype datasets are reported in different columns, while the involved host species are reported in the cells.

The evaluation of the isoelectric point of the amino acids detected by PRIME (**Supplementary Figure 1**) or involved in HS binding (**Supplementary Figure 2**) revealed that, although an overall stability featured most sites, some showed more intense fluctuations. Overall, PCV2b and d revealed higher value compared to PCV2a, with the exception of position 72, 88 and 131. Strain circulating in domestic vs wild animals showed a comparable tendency, although the lower number of available sequences in wild dataset and the scattered distribution through time and space led to more marked fluctuations and less predictable patterns. Interestingly, PCV2a and d experience more marked variations over time, with opposite tendencies, directed toward a decrease in PCV2a and in increase in PCV2d dataset.

## 4 Discussion

PCV2 is characterized by remarkable genetic and, to a lesser extent, phenotypic variability. Different genotypes have emerged and have been identified: among those, PCV2a, PCV2b, and PCV2d are the most successful, demonstrating temporal persistence and worldwide distribution. Even within these major genotypes, significant heterogeneity has been observed, and distinct epidemiological patterns have occurred, with the early emergence of PCV2a, followed by PCV2b and PCV2d. However, recent studies have revealed the persistent circulation of all these genotypes, with periodic minor fluctuations rather than complete replacement, as initially thought (15). Virulence and immunological determinants, along with managerial factors, have been suggested to play a role in shaping the emergence of PCV2 and its complex epidemiology (4, 44, 45). Nevertheless, how these different determinants acted and interacted has never been investigated from a historical perspective. Since the environment is the main determinant of any organism’s fitness and evolution, from a viral perspective, the host effect must clearly be considered. While PCV2 was initially —and still is — most frequently identified in intensively raised domestic pigs, a high prevalence has also been reported in wild boars. Accordingly, structured coalescent analysis inferred a wild boar origin of PCV2, followed by its introduction into domestic pigs potentially centuries ago. Even if plausible, it must be stressed that inferences of such ancient events, based on sequences available only from the last decades, must be taken with caution, while proper consideration must be devoted to recent periods only. Interestingly, a high wild-to-domestic viral migration rate was estimated in this study, which contrasts with previous reports from Italy (24). Several explanations are possible. First, different epidemiological scenarios may determine varying contact opportunities and viral flow. In regions where farming systems have lower external biosecurity measures, facilitating intense pig–wild boar interactions—either directly or through rural farms—there may be an increased introduction of “wild” strains into the domestic sector. Alternatively, the different evolutionary scales (country vs. global) may play a role. In the Italian scenario, analyses were likely based on a shorter time frame, limited to recent decades when the intensive pig sector was already well-developed. In this context, the overwhelming presence of domestic viral populations may have made the influx of wild strains appear less significant. In contrast, the present study was able to model the early phase of PCV2 circulation and inter-host dispersal, a period when host population structures and management systems were completely different. A combination of both hypotheses—differences in temporal scale and management systems—cannot be excluded. This suggests the usefulness of conducting additional country-based analyses to further investigate the determinants and directionality of viral fluxes in different regional contexts. As expected, the viral population hosted by domestic pigs was an order of magnitude larger than that in wild boars, regardless of the considered genotype.

Such condition generated a huge effective viral population size, which is a fundamental prerequisite for viral evolution to occur. Moreover, PCV2 introduction and circulation in intensively raised and managed pig population likely posed stronger, pervasive and more homogeneous selective pressures, with the immune-induced one being considered the driving one. Most of the positions detected under positive selective pressure were exposed on the capsid surface and more particularly in regions that were experimentally proved as epitopes (36–41). Variation in several of these amino acids was proven to impair or decrease the binding affinity of monoclonal antibodies, thus likely affecting the cross-protection among strains and genotypes. Among these amino acid positions, 30, 59, 63, 89, 130, 131, 133, 190, 191, 206 and 210 (46, 47) were detected by MEME and/or 206–210 by FADE also. While some positions were identified as under episodic diversifying selection across most datasets—suggesting a dominant and shared role in immune response and evasion (e.g., amino acids 59, 133, 134)—others were detected only in specific viral groups or hosts. This evidence highlights the complex network of interactions driving viral evolution, potentially influenced by the unique characteristics of the host populations in which the virus circulates (e.g., strain exchange, pre-existing immunity, etc.), as well as the overall capsid structure and interactions among different regions. Notably, a significantly higher number of sites under selection were detected in datasets of strains circulating in domestic pig populations. The strong immune pressure in these populations, resulting from both high viral circulation and, more importantly, widespread vaccination, might explain the intensified selection pressures (19). These findings may contribute to a better understanding of viral evolution in response to extensive vaccination and, ultimately, to the development of new strategies targeting dominant epitopes or regions characterized by lower flexibility and evolutionary potential. Comparing selective pressures site by site revealed that viruses in wild boars experience generally higher pressure than those in domestic pigs—except at certain surface-exposed sites. This pattern likely reflects the high genetic variability and diverse host environment in wild boars, which drives broader diversification, whereas the more uniform immune response in domestic pigs, influenced by widespread PCV2a vaccination in a high-turnover population, intensifies positive selection at specific sites Supporting this scenario, several capsid positions appeared under directional selective pressure, indicating a tendency toward specific amino acids following the virus introduction into domestic populations from wild ones. The two host populations can thus be viewed as distinct environments where different phenotypes exhibit varying fitness optima. Although such analyses might be biased by the ‘founder effect’—where a single individual with a particular phenotype gives rise to an independent clade—in the PCV2 scenario observed here, and supported by structured coalescent analysis, strains collected from wild and domestic populations were interspersed in the phylogenetic tree, with multiple independent introduction events inferred. In fact, for the same capsid position, multiple ‘evolutionary solutions’ toward which selection was directed were identified, indicating the evolutionary plasticity of PCV2. Comparable to observations in the analysis of diversifying selection, the majority of these positions were exposed on the capsid or viral surface, implying the role of immune pressure. However, the evaluation of substitution patterns also highlighted a tendency in most of the considered positions to preserve or increase amino acid polarity. In several instances, neutral amino acids were replaced by polar or charged ones, and polar or charged residues, when substituted, were replaced by other polar or charged residues, although some positions exhibited different patterns. The PRIME analysis, specifically designed to evaluate mutations leading to radical changes in amino acid features, was performed with a focus on amino acid isoelectric points and confirmed this scenario for several residues. Variations in charge at key positions can affect the virus’s capability to interact with host proteins (48). For PCV2, Dhindwal et al., revealed the areas involved in binding HS and identified key amino acids in positions 58, 59, 63, 73, 89, 129, 132, 186, 188 and 222 (42). HS proteoglycans are ubiquitously expressed on most cell surfaces. Due to their highly sulfated glycosaminoglycan chains, they carry a negative charge that can electrostatically interact with the basic resides of viral surfaces. PCV2 binding to T-lymphoblasts has been reported to be partially mediated by chondroitin sulfate (CS) and dermatan sulfate (DS). Studies have shown that T-lymphoblasts from Landrace pigs are more susceptible to PCV2 infection compared to those from Piétrain pigs (49). This increased susceptibility was associated with differences in viral replication kinetics and the role of glycosaminoglycans (GAG) in viral entry (49). Similarly, phosphacan, a glycoprotein containing several CS chains, has been implicated in PCV2 attachment and internalization in monocytes. Research indicates that monocytes from Piétrain pigs exhibit a higher capacity for PCV2 uptake and degradation compared to those from Large White and Landrace breeds (43, 50). These findings suggest that variations in GAG-mediated viral entry and host cell processing contribute to the observed differences in PCV2 susceptibility among pig breeds, which involve the variable expression of GAG among them. Genetic factors artificially selected over time might thus have affected the susceptibility of the host population. Simultaneously, variability among PCV2 strains uptake capability was observed, particularly in the distribution of positive charges on the capsid (43). This variability creates the prerequisite for this characteristic to be subject to differential selection pressures, potentially influencing viral fitness.

In the present study, we observed selective pressures favoring changes in the capsid charge profile across all PCV2 genotypes, particularly in those circulating among domestic pigs, with PCV2d showing the most pronounced alterations. In contrast, strains circulating in wild boars exhibited fewer similar changes. Studies comparing gene expression profiles between wild boars and domestic pigs have revealed differences in the expression of genes involved in immune responses and viral recognition. These variations may have prompted the virus to develop compensatory strategies to enhance its fitness in domestic pig populations, including an increase in attachment and internalization capability. Most of the inferred sites were, in fact, located at or near positions known to be involved in HS-like molecule binding, as proposed by Wei et al., 2019 (51), and Ouyang et al., 2023 (43), affecting receptor binding and PCV2 uptake. Notably, several of the amino acids identified in this study are part of the ‘three-wings windmill’ and the pentagon pattern of positively charged residues whose presence and variability were suggested to influence the differential binding capabilities of various strains (43). The evaluation of charge of the critical amino acid over genotypes, host and time showed variable patterns. The overall tendency of PCV2b and PCV2d, particularly in domestic pigs, to have or point toward a higher isoelectric point in specific position (e.g. 59, 63, 68, 133, 134, 136, etc.) in recent years suggests an adaptative variation which may lead to a more efficient binding to the negatively charged GAG receptors, as previously suggested in the Belgian scenario (51). However, exceptions were also noted suggesting the involvement of complex patterns. The impact of enhanced HS binding on viral fitness varies among different viral species. In some cases, increased affinity for HS facilitates viral attachment and entry into host cells, thereby enhancing infectivity. Conversely, for other viruses, heightened HS binding can lead to reduced virulence or dissemination capabilities (48). This variability underscores the complex role of HS interactions in viral pathogenesis. It can be speculated that also for PCV2 an increasing binding capability might lead to both increased infectivity, or uptake and degradation, depending on other viral or host factor (43, 49, 51, 52). Therefore, the trade-off associated with an increase in positive charges at critical capsid positions may depend on the environment in which the virus circulates. The contrasting trends observed among genotypes might represent the results of different evolutionary strategies. Low-level persistence or more aggressive replication strategies might be both favored from an epidemiological perspective, depending on factors such as host species, geographical distribution, and farm management practices (including pig breeds). These variables may contribute to the complex patterns observed and warrant further investigation, including experimental studies. Additionally, the same positions involved in HS binding are also targeted by the host immune response, creating overlapping pressures acting on these sites. The directionality and magnitude of these pressures are difficult to disentangle, adding further complexity to the proposed evolutionary model.

## 5 Conclusions

The present study investigates the complexity of evolutionary forces shaping PCV2 phenotypic and, indirectly, genotypic variability. Amino acid positions exposed on the viral surface exhibited a higher tendency for diversification, suggesting that immune-induced selective pressure and variations in receptor-binding capability due to amino acid charge differences at specific capsid positions contribute significantly to viral evolution. A combination of these factors is likely at play, although their relative influence remains challenging to determine. Moreover, the variability observed between viral populations circulating in wild boars and domestic pigs confirms the role of the host environment as a major driver of viral evolution. This observation provides insights into how viral introduction into domestic animals and the intensification of farming may have contributed—and continue to contribute—to PCV2 evolution, and potentially that of other viruses as well, influencing the current epidemiological and clinical scenario. It is important to acknowledge that the study is not without limitations. The use of convenience sampling and biased sequence availability, for instance, may yield a sample that is not fully representative of the populations under study—particularly as wild boars sequences are underrepresented and less homogeneously distributed on a global scale. Nevertheless, the analytical approach employed helps to mitigate some of these sampling biases. Additionally, incorporating data from rural animals, for which sequence information is extremely limited but whose epidemiological role has been largely proven, could provide a more comprehensive understanding of the relative impact of farming systems versus natural host environments. As a future perspective, validating and refining the large-scale epidemiological findings of this study in experimental or more controlled settings would be of considerable interest.

## Data Availability

The original contributions presented in the study are included in the article/**Supplementary Material**. Further inquiries can be directed to the corresponding author.

## Supplementary material

Supplementary Figure 1 Trends in amino acid isoelectric point variations over time, calculated using the LOESS function as implemented in ggplot2. Each panel represents statistically significant amino acids identified by PRIME analysis. Different genotypes and hosts are distinguished by various colors and line types. Shaded areas represent the 95% confidence intervals, calculated through bootstrap analysis.

Supplementary Figure 2 Trends in amino acid isoelectric point variations over time, calculated using the LOESS function as implemented in ggplot2. Each panel represents amino acids identified by Dhindwal et al., 2019 (42) as involved in heparan sulfate binding. Different genotypes and hosts are distinguished by various colors and line types. Shaded areas represent the 95% confidence intervals, calculated through bootstrap analysis.

Supplementary Table 1 List of sequence accession number used in the present study and relative metadata. Pairwise genetic distance plot of domestic pig and wild boar sequences is also reported.
