# Supplementary figures and images for: Host-driven evolution of PCV2: insights into genetic diversity and adaptation
Source: Front Immunol. 2025 May 26;16:1577436. doi: 10.3389/fimmu.2025.1577436 (PMC12146169; doi:10.3389/fimmu.2025.1577436)

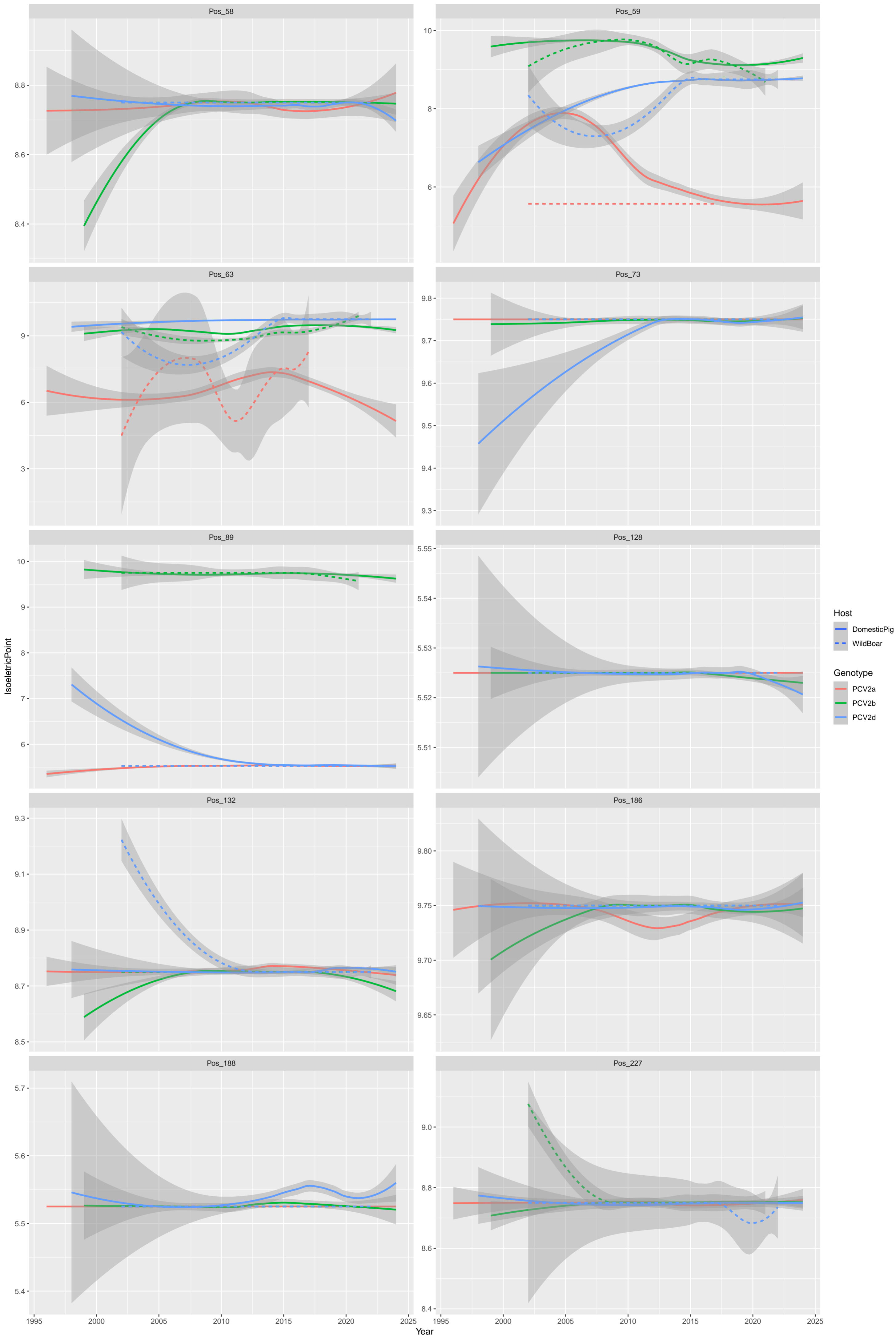

Supplement: Supplementary Figure 1 — Trends in amino acid isoelectric point variations over time, calculated using the LOESS function as implemented in ggplot2. Each panel represents statistically significant amino acids identified by PRIME analysis. Different genotypes and hosts are distinguished by various colors and line types. Shaded areas represent the 95% confidence intervals, calculated through bootstrap analysis. [file DataSheet1.zip › Supplemetary figure 2.pdf]

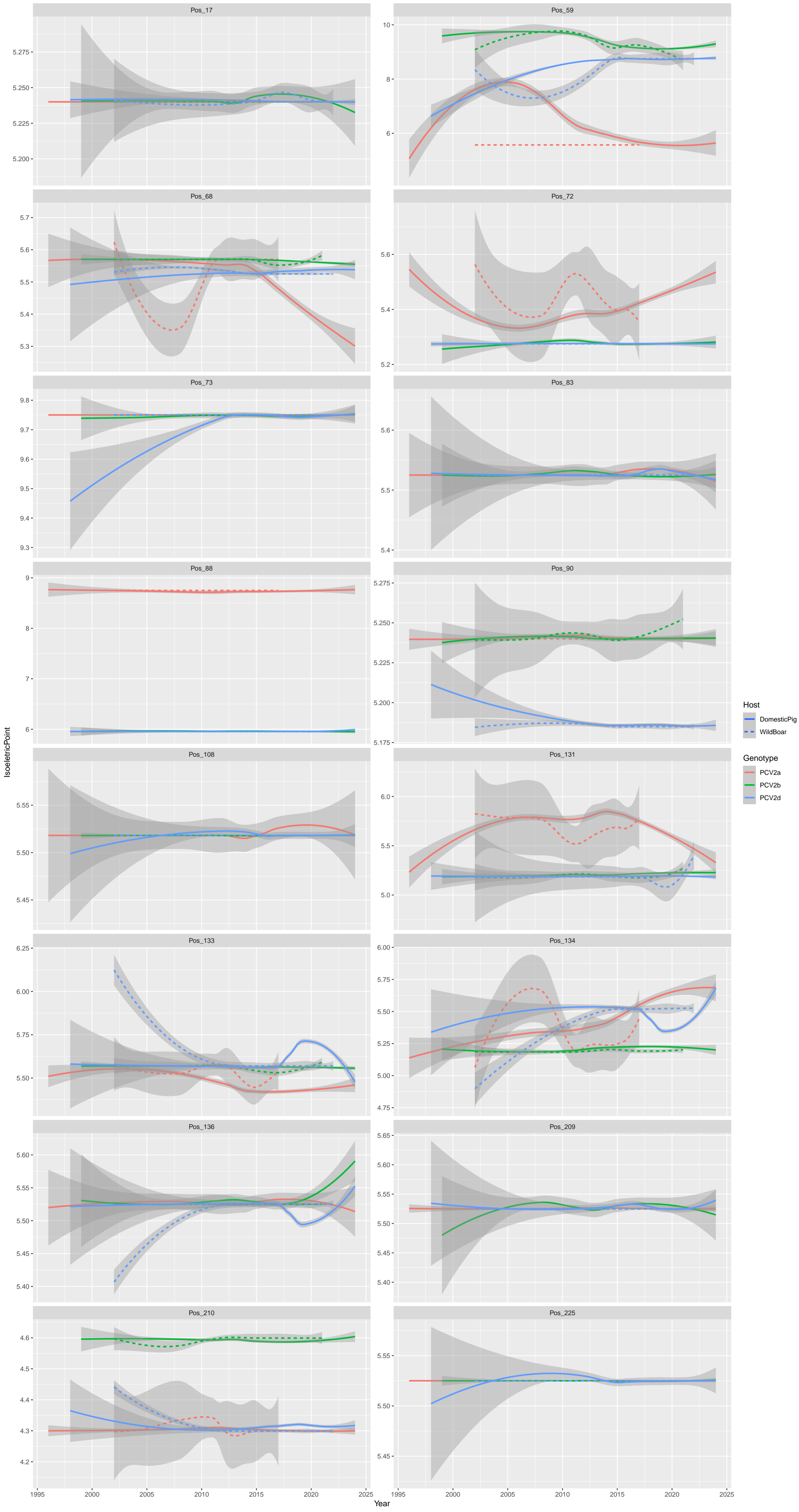

Supplement: Supplementary Figure 1 — Trends in amino acid isoelectric point variations over time, calculated using the LOESS function as implemented in ggplot2. Each panel represents statistically significant amino acids identified by PRIME analysis. Different genotypes and hosts are distinguished by various colors and line types. Shaded areas represent the 95% confidence intervals, calculated through bootstrap analysis. [file DataSheet1.zip › Supplemetary figure 1.pdf]
